# An Inquiry into the Importance of Physical Culture as a Branch of the Science of Prevention

**Published:** 1888-06

**Authors:** Wm. C. Bailey

**Affiliations:** Albion, N. Y.


THE BUFFALO MEDICAL AND SURGICAL JOURNAL

VOL. XXVII. JUNE, 1888. NO. 11

### Original Communications.

### *AN INQUIRY INTO THE IMPORTANCE OF PHYSICAL CULTURE, AS A BRANCH OF THE SCIENCE OF PREVENTION.*

BY WM. C. BAILEY, M. D., Albion, N. Y.

GENTLEMEN—The consent to address you to-day was given by me with much hesitation. Not presuming that I might be able to present anything new to such an audience, the hope was cherished that, informally, something might be said to arouse an interest in a subject of growing importance, and prompt further inquiry.

Medical education has, within recent years, steadily progressed; and the faculty and alumni here to-day have just cause for congratulation upon the standard this college has taken in promoting such progress.

But there is another step in advance the future must bring:

We have yet to learn more fully the truth, that the first and grandest obligation the physician owes society and the state is to prevent disease.

We are almost compelled to recognize this advancement, on account of the wondrous progress made in every branch of the medical art. As a result of etiological research, many causes of disease have been discovered that are now known to be of a preventable nature. And we are beginning to believe, so limitless has been this progress, that, as was recently asserted, there is no physiological reason why life may not be extended indefinitely, provided it were possible for us to comprehend fully and obey implicitly all of nature's laws. Understanding preventive measures to be those which are employed to anticipate disease, and the science of prevention (I do not like that term preventive medicine), as that which concerns the health of the people, it must be conceded that a most essential requisite to perfect health is a well-developed mind and body.

The fact that the tendency of the present age has been almost exclusively to encourage and popularize mental culture, the body only valued as a necessary support of the mind, is sufficient reason to present a subject of this character for your consideration.

The sensibilities of the mind and body are so acute, the fact that the one, either in health or disease, is continually modifying the condition of the other, hardly needs demonstration.

If this be true, it follows that "the highest permanent perfection of the mind may only be found with the most healthful state of the body."

The question then naturally suggests itself: What are the best means to be employed in producing such development?

But before answering, it is indispensable that we comprehend somewhat the nature of this mental and physical co-relationship.

This leads us into metaphysical fields; and the further we extend our studies, the more intricate seems to be the connection, until finally, we are led to exclaim, "Where is the chasm that separates them?"

Does that which we term mind extend to and include the nerve ganglia in remote parts of the body; or are its limits confined within the boundary of the gray matter of the brain and spinal cord; or is it held within that mass that fills the bony vault; or even here may it only embrace those portions that are the seat of the highest attributes, as ideation, perception, volition?

Self's communication with things external is by means of various movements. These are indicative of what is termed life, and signify a higher or lower degree of intelligence.

In their simplest form, these movements may be represented by two nerve fibers, connecting with each other through or by a nerve cell or set of cells. One of these fibers conducts an external impression to the nerve center. This is instantly transmitted to the other fiber, and motion is produced by contraction or relaxation of the appropriate muscular tissue. For example, in passing through a gallery of paintings, we are suddenly attracted toward a special work of art. The impression received, conveyed to the proper nerve centers, causes us to cease our walking and gaze intently upon the picture. Our own knowledge of this impression is by the movements resulting therefrom—our attitude of silence, our fixed attention. This is a conscious deed, and betokens more or less intelligence.

There are numberless other acts, seemingly performed without our knowledge. In fact, one upon reflection is surprised to learn how large a part of his existence consists of acts requiring scarcely an effort of perception or volition.

The merchant goes to his place of business. His mind is absorbed with anticipated cares. He, however, avoids running into trees or posts, salutes a passing friend, and turns the proper corners on the way. He does not appear to be conscious of these acts; but let him close his eyes, and his steps would occasion confusion, and doubtless injury.

Now, these varied movements may result from heredity, as is instanced in the characters portrayed in the writings of Alexander Dumas; from instinct, all animals displaying beautiful illustrations of this, but, notably, the bee and the ant; from acquired habit, an automatic-like impression (if I may use that term), produced upon certain nerve centers, as the result of frequent repetition of the same act, until finally it becomes inherent, "and it is the desire of the organism to display that function which is embodied in its nature;" and, finally, from an intuition or cognition implanted within us. This is not wholly instinct, for unlike the latter, there may be improvement in its method. For instance, we intuitively avoid that which, from experience and not from instinct, we know to be injurious.

We have been considering movements resultant from impulses received from things about us.

There are certain indications of the inner processes of the body and mind; and these are made manifest by what is termed *expression.*

The body exists under certain conditions. Expression is the outward indication of these conditions.

The eager manner in which a child may grasp a glass of cold water, expresses a certain condition of the body we denominate thirst.

Like the movements that concern external stimuli, the variety of expressions is great: the motions of the body, the movements of the hands, the poise of the head, the pitch or tone of the voice, the muscles of the face in their varied attitudes, the color of the cheeks.

Most of these movements and expressions are subject to development, and may be improved by education. In truth, it is in the undeveloped being we find the most expressionless manifestations; and it is in the perfected manhood we easiest detect character. Here every line seems to display some emotion or feeling. It is thus we read thought. It is the mind visible. This the poet must have had in view when he wrote:

> "Her pure and eloquent blood
> "Spoke in her cheeks, and so distinctly wrought,
> "That one might almost say her body thought."

The greater the development of these inner conditions by expression, then, the greater the intelligence. The paralytic, or the imbecile, may present an almost entire want of this beautiful and useful provision of nature.

Now, may we not ask, "Where is the seat of responsibility of these movements and expressions?"

In the gradation of animal life, the fish is the first where there is known to be a connection between nerve fibers and sensory ganglia. In the scale below, the brain wholly absent, impressions are received and movements produced, showing that at least in a certain proportion of the animal kingdom, this organ is not required to originate action.

As we ascend the scale, we find the brain gradually is extended in size, and, finally, also in convolution, "through the amphibia, birds, mammalia, higher monkeys, to man; and in the ascent we discover, of course, a gradual development of intelligence.

From the interesting study ot these animal gradations which we have thus briefly reviewed, it seems clearly proven, by experiment oft repeated, that not only remote from the brain, but also outside of the spinal cord, are nerve-ganglia originating certain acts, especially of our organic life; and that, located in the spinal cord are functions especially important. Thus the cord itself is not only a conducting organ between external things and the brain, but also contains many independent nerve centers (Maudsley). A large portion of the acts to which we have directed attention, are dependent upon these ganglia for guidance. Pfluger's experiments serve well to emphasize this important point. He pinched the hind foot of a decapitated frog, and it immediately made efforts to draw the foot away. The pressure increased, caused the frog to redouble its efforts. These failing, there was a muscular twitching for a moment. Finally the other foot was used to force the obstruction away. This teaches us that the impression implanted upon the ganglionic centers, called into action the corresponding nerves of the opposite extremity. But this failed also. After a brief period other sets of ganglia seemed to be impressed, and with one convulsive effort the animal leaped away. Here was "an adaptation of means to an end in a headless frog," with an entire absence of its center of nerve force—that organ where is alone supposed to originate movements that have a definite purpose.

To illustrate further, we are reminded of the discussion which D'Israeli says occurred between Cecco and Dante upon natural and acquired genius. The former claimed that nature is more potent than art, while the Italian poet asserted the contrary, and cited, as an example, that his cat had been taught to hold a lighted candle while he supped and read. He requested Cecco to call and witness the experiment. The invitation was accepted. While the experiment was in progress, the guest suddenly let go some mice he had concealed. The cat immediately forgot its acquired genius and leaped toward the escaping rodents.

"The anencephalic child exhibits movements of its limbs, and cries and coughs."

Darwin's observations upon the manifold motions and expressions of the healthy infant, during its developmental period, are invaluable. To them I can only call your attention.

Are these examples the results of the highest functions of the intellect, or may they be due to reflex action, stimulated by nerve ganglia distant from the brain?

If either, we may inquire with Maudsley, are they mental or physical? If mental, we must enlarge our conception of the mind, and acknowledge it is not wholly confined to the limits of the brain, but includes, as well, portions of the spinal cord, or perchance even ganglia separate from this. If physical, then must we elevate our idea of the value of the body, and deduct functions popularly supposed to belong alone to mind.

We have dwelt thus at length, but very imperfectly, upon these things, to indicate how intimately related are body and mind, how interdependent they are, and how difficult it is to determine the line that separates them. This has seemed requisite, that we may appreciate the importance of caring for the body if we would expand the mind; that physical culture is essential, if we would reach a high mental development. In truth, we must assent, that, to define what and where mind is, and to explain the length, and breadth, and height, and depth, of its relationship with the body, is as much beyond our comprehension as it is for that little animal whose life continues but an hour, to appreciate the geography of a continent.

Our next inquiry here arises: How may perfected manhood be attained? We answer, briefly, by exercise. This, in its liberal and proper signification, means education, labor and recreation, in all their forms. What, to-day, is education, if not a series of graded mental and physical exercises? And what is labor, if not an activity of brain and muscle to accomplish a certain end?

"The frame of every individual," says Maclaren, "bears within itself the germ of its own perfectibility." Now the means whereby such an end may be attained, in its growth and development, is *exercise*, without which life cannot exist. It begins at the cradle, exemplifies itself during the period of growth, and a constitution is formed upon its faithful continuance. This should characterize adult life, in order to preserve the constitution thus formed, for it is that which makes the man what he is.

We need not, to-day, discuss the sort of exercise essential to one's well-being. This varies with and in each individual.

These general propositions, however, should not be disregarded—

First. Beginning with early childhood, there should be a gradual unfolding—an evolution—in the forms of activity, as the system itself grows and unfolds its higher purposes.

To this end, children should be taught habits of exercise and industry, acquired habits, as we have attempted to define them. These should begin with their A B C's, and grow up with them. The system of school education would be advanced, were students, in order to receive their promotion from any grade, required to attain a certain standard of physical improvement, properly proportioned to age, constitution and history; and that they may receive their diploma at graduation, were they expected to possess at least a portion of the physical qualifications demanded by the government to enter West Point or Annapolis. This would popularize the development of physical culture, produce a hardier race, materially lessen sickness, extend life, improve the morále of the people, augment mental capacity, and add wealth to the nation.

Again, to produce harmonious growth, this activity should be of such a character as to develop, and be continued in every part, both of mind and body.

It is this which attains to the name of beauty. In disproportionate development, however slight, there is deformity. This was the Greek conception of beauty—the absence of all deformity. What a complete description? Did you ever think of it? If attempting a sketch of a human figure, do you not paint a perfect one if you deform no muscle, no expression, no organ? If the head be too large, or the chest too narrow, or the nose too rounding, in proportion with the rest of the figure, you have deformity and have not beauty. Can such perfection as the Greeks conceived, and the Creator intended, be realized in the development of mental power and the neglect of the physical, or in the development of the physical and the neglect of the mental?

In either instance, would you observe those expressions depicted upon the face, and in the motions and attitudes of the body characteristic of highest culture? Do you see portrayed in the face of a Sullivan those lines found in complete manhood? Is there not rather a want of expression, as noticeably belongs to the criminal and uneducated classes?

Finally, the end to be accomplished, if we understand these principles aright, is our highest good.

This is health.

Where every part performs perfectly its end, health exists, and love, and happiness, and the other higher moral attributes of our nature, follow.

Disease, suffering, and misery cannot enter here so long as this condition shall continue. When we see man in this, his perfect state, we must acknowledge he exists for something more than selfish ends. In him is developed perfect unity of body and mind, producing harmony and satisfaction, well worthy of the poet's description: "So noble in reason; so infinite in faculties; in form and moving, so express and admirable; in action, so like an angel; in apprehension, so like a God."

